# Effect of different pacifier designs on orofacial tissues: a computational simulation comparative study

**DOI:** 10.1007/s00784-025-06428-9

**Published:** 2025-06-25

**Authors:** Rita Pereira, João Romero, C. P. Santos, Ana Norton, João Miguel Nóbrega

**Affiliations:** 1 IPC – Institute for Polymers and Composites, University of Minho, Azurém Campus, Guimarães, 4804-058 Portugal; 2 CMEMS – Center for MicroEletroMechanicsSystem, University of Minho, Azurém Campus, Guimarães, 4804-058 Portugal; 3 FMDUP – Dental Medicine School of University of Porto, Porto, 4200-393 Portugal

**Keywords:** Computational modeling, Ergonomic design, Malocclusions, OpenFOAM, Pacifier, solids4Foam

## Abstract

**Objectives:**

This study analyzes the effects of different pacifiers on the malocclusion formation, emphasizing the significance of pacifier design. Based on a computational model of pacifier sucking developed by the authors, the study provides insights dependent on pacifier geometry.

**Materials and methods:**

A computational model was developed, consisting of the palate, pacifier, and tongue, including six tissues: mucosa, cortical bone, cancellous bone, and alveolar bone, periodontal ligament, and teeth. Three types of pacifiers were analyzed: orthodontic, standard, and conventional. The geometries were obtained from manufacturers and modeled using Blender™ software. The model was implemented in the OpenFOAM^®^ library to calculate tooth displacement, exerted force, and stress distribution on the palate tissues.

**Results:**

The results provide a clear comparison between pacifier models, showing that well-designed pacifiers, Orthodontic (OP) and Standard (SP), are significantly less harmful to oral structures than the Conventional Pacifier (CP). OP and SP reduced the volume of the region subjected to high von Mises stress (0.05–0.01 MPa) on the palatal mucosa by 95.70% and 93.95%, respectively, when compared to CP. Furthermore, they led to a maximum reduction in mean tooth displacement of 79% (OP) and 75% (SP). These findings indicate that the pacifier design can significantly impact mechanical loading on the palate and teeth, reducing the risk of developmental oral malocclusions.

**Conclusions:**

This study underscores the importance of pacifier design in mitigating potential adverse effects on orofacial development.

**Clinical evidence:**

There is a growing need for pacifiers designed based on scientific evidence to reduce the risks of orofacial deformation resulting from non-nutritive sucking. The computational approach introduced provides valuable insights that can inform the design of improved pacifiers aimed at minimizing risks associated with non-nutritive sucking habits. It is hoped that this method will guide the future development of more effective pacifiers, reducing potential adverse effects on orofacial structures.

**Supplementary Information:**

The online version contains supplementary material available at 10.1007/s00784-025-06428-9.

## Introduction

### Prevalence and clinical impact

Non-nutritive sucking (NNS) habits refer to sucking behaviors unrelated to feeding, such as pacifier use and thumb sucking. The pacifier is one of the most common forms of NNS among young children, with prevalence ranging from 13% to nearly 100% at some point during childhood [1]. While this behavior is considered normal in early childhood, its persistence may lead to changes in orofacial development.

The prevalence of non-nutritive sucking habits varies between 17.7% and 90.7%, depending on the population studied [2, 3]. By 12 months of age, it may reach up to 42.5% [4], but it tends to decrease over time, with most children discontinuing the habit by around 3.5 to 4 years of age [2, 5].

Several studies and literature reviews indicate a strong likelihood that non-nutritive sucking with a pacifier causes various types of malocclusions, such as anterior open bite (AOB), posterior cross bite (PCB), increased overjet, upper incisor protrusion, and decreased superior intercanine width, which are major changes in primary dentition [2, 3]^6^]- [25].

Evidence suggests that non-nutritive sucking habits lasting more than six hours per day can significantly influence the resting posture of oral structures, particularly the tongue, thereby contributing to the development of malocclusions [26].

### Biomechanical considerations

The first studies on the prevalence of malocclusions across different populations date back to the early 1900s [7, 10]. However, it was only in the 1980s that the relationship between prolonged pacifier use and occlusal alterations began to be systematically investigated. Since then, numerous studies have reported that 39–93% of children exhibit some form of malocclusion [10], with prevalence ranging from 38 to 94% among those with sucking habits [11].

Malocclusions refer to dental misalignments or discrepancies between the jaws, which may have genetic, congenital or environmental origins. Common types include anterior open bite (AOB), posterior crossbite (PCB), increased overjet, deep bite and narrowing of the upper arch [26, 27]. Among environmental factors, pacifier use significantly impacts orofacial development [27].

The specific type of malocclusion that develops depends on several factors, including the pacifier’s shape and position, associated orofacial muscle activity, mandibular posture during sucking, the child’s genetic predisposition, and the intensity, frequency, and duration of the applied forces [28].

AOB has been reported in up to 96.3% of pacifier users, though it occurs less frequently with orthodontic models [8, 29]. PCB affects 12.8–88.9% of children and shows no significant reduction with orthodontic pacifiers [8]. An overjet greater than 4 mm occurs in 17.79–28% of pacifier users, compared to only 4% in non-users, while an overjet greater than 2 mm is observed in up to 67.5% of cases [8, 10]. Overbite, AOB, and PCB are also more common among long-term users [10, 30].

Duration of use is a key factor: the risk of occlusal changes increases after 24 months and becomes more pronounced beyond 36 months [8, 29]. One study of children in the mixed dentition phase found that 55% exhibited AOB, bilateral PCB, increased overjet, and overbite, linked to prolonged pacifier use and thumb sucking beyond 60 months of age [30].

Discontinuing the habit between ages 3 and 4 is associated with a higher likelihood of spontaneous correction, particularly for AOB [6, 8], while continued use may negatively affect permanent dentition [31].

Nevertheless, the literature remains inconclusive, and the effects of pacifier use on primary dentition have not been definitively established.

### Previous computational work

Computational modeling has been employed to investigate the mechanical effects of pacifiers on oral structures; however, many studies still rely on simplified representations. Levrini et al. [32] analyzed different pacifier designs using a virtual oral model, but lacked sufficient detail regarding the applied parameters, which limited the reliability of their findings. Subsequent studies [33, 34] introduced more anatomically accurate reconstructions of the palate and tongue, revealing that orthodontic pacifiers tend to generate higher palatal stresses and affect incisor positioning, whereas models such as the Super Soothie™ promote a more favorable stress distribution and stimulate maxillary growth. Nevertheless, these studies do not adequately account for tissue properties or the dynamic nature of the sucking process.

More recent models [35, 36] have aimed to represent the suction cycle and pacifier-palate interaction with greater fidelity, incorporating hyperelastic materials and additional tissue layers. These studies demonstrated that pacifier design and size directly influence the stress and deformation distributions on the palate. Despite these advancements, limitations persist regarding the full anatomical representation of the oral cavity and the replication of real physiological conditions [37–41].

In this context, the present study proposes a comparative analysis of different pacifier designs using a previously validated computational model, fully detailed in the authors’ earlier work [42], to provide a more accurate assessment of the biomechanical effects on the infant palate.

### Rationale for this study

Manufacturers aim to develop pacifiers that soothe infants while minimizing potential adverse effects. However, pacifier design methods largely rely on empirical knowledge and assumptions, making it difficult to experimentally measure their impact on the palate and dentition. Consequently, a variety of pacifier types are available, differing mainly in nipple shape. The most common pacifiers can be categorized into two main types: conventional and orthodontic. Orthodontic pacifiers feature a convex curvature that conforms to orofacial structures, particularly the palate [43], while conventional pacifiers have a cherry-shaped nipple and are generally thicker than their counterparts [43]. Although both the orthodontic pacifier and the standard pacifier have similar external geometries, the orthodontic pacifier is thinner where it contacts with the palate. Some studies suggest that orthodontic designs better adapt to the child’s oral anatomy [32, 44–46], but due to the limited ability to quantify their effects, conclusions remain inconclusive.

Previous studies have primarily focused on either non-nutritive sucking habits or pacifier design in isolation. Most adopted qualitative approaches and do not precisely quantify the mechanical forces exerted during sucking [47]. This limitation highlights a lack of ergonomic perspective in product design [18, 19], making it difficult for manufacturers to base design decisions on technical evidence [47].

Moreover, current research does not assess the combined effects of sucking behavior and pacifier design, limiting a comprehensive understanding of the biomechanical mechanisms involved and undermining the effectiveness of preventive strategies.

This work proposes and implements a computational approach capable of simulating non-nutritive sucking with a pacifier, enabling the quantification of its effects on orofacial structures, specifically on tissues related to the development of malocclusions [42].

In this study, the validated computational model is applied to compare the effects of different pacifier designs. The model allows, for instance, the assessment of palatal stress distribution and the force transmitted to the teeth, depending on the pacifier type. These results enable a quantitative comparison of various designs under identical sucking conditions, allowing inference of their potential to induce malocclusions and quantification of the severity of such outcomes.

The developed computational tools and insights gained are expected to support the future design of innovative, higher-performance pacifiers.

## Materials and methods

### Computational model composition

**Fig. 1:**
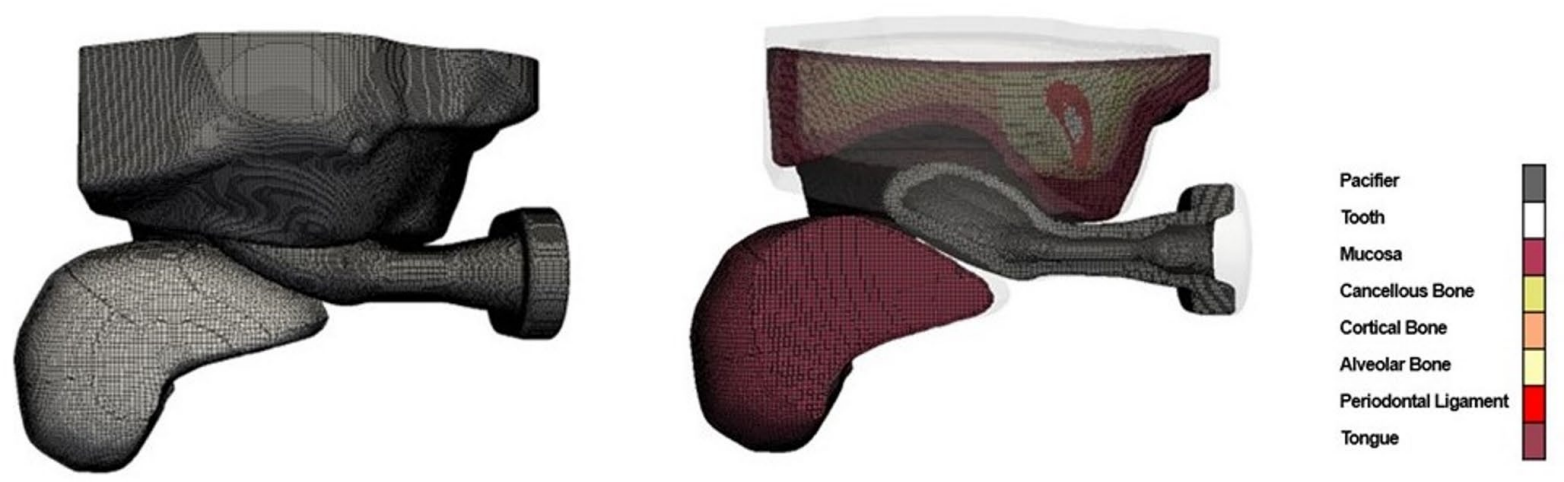
3D outer full computational mesh of the orthodontic pacifier model, generated using the cfMesh tool [54] (left), and cut view showing the defined cell sets (tissue regions) (right)

In a previous work, an innovative computational based pacifier assessment methodology (Fig. 1) was developed and evaluated [42]. The detailed methodology is described in Pereira et al. [42], while the specifications of the computational model are summarized in the Supplementary Information.

The implemented approach consists of a computational methodology capable of providing quantified data on the effects of pacifiers on the oral cavity, thus inferring the relationship between the pacifier and the prevalence of malocclusions. The results obtained clearly complement the empirical tests presented in the literature regarding the effects of different pacifiers on orofacial structures. The computational model comprises a palate, a pacifier, and a tongue. The palate model, obtained by scanning a physical plaster cast of a six-month-old infant’s palate [42], includes six tissues: mucosa, cortical bone, cancellous bone, alveolar bone, periodontal ligament, and teeth.

The 3D outer and cut views shown in Fig. 1 showcase the employed computational mesh. Moreover, the last allows the identification of the computational cells corresponding to all pacifier tissues and tongue.

**Fig. 2:**
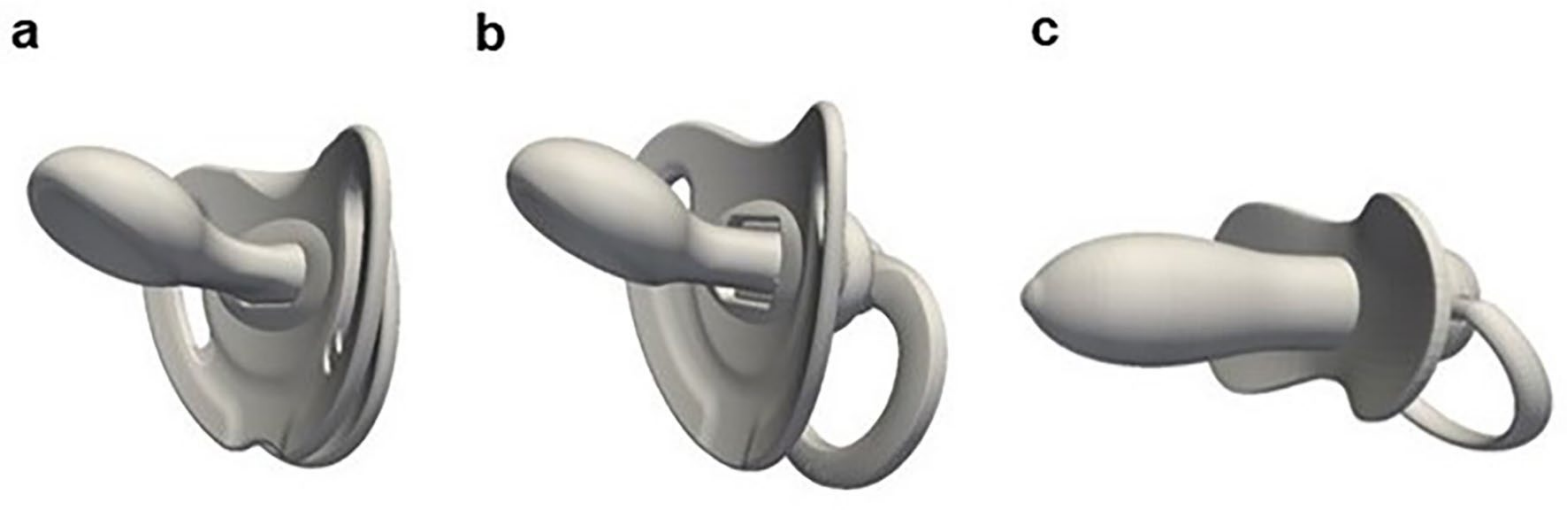
3D complete models of the tested pacifiers: **a**. Orthodontic Pacifier; **b**. Standard Pacifier; **c**. Conventional Pacifier

In the present work, three different pacifiers, representative of the wide range of products available on the market, were analyzed: an orthodontic pacifier, a standard pacifier and a conventional pacifier, all illustrated in Fig. 2. The geometries of the orthodontic pacifier and the standard pacifier were provided by the manufacturer, while the conventional pacifier was modelled based on measurements taken from actual models using the 3D modelling software Blender™ [48]. These specific pacifier models were selected based on prior studies available in literature, which also compare the orthodontic and conventional designs [32–36]. Furthermore, the inclusion of both the orthodontic and standard models in the present study aimed to assess whether minor design optimizations in pacifiers could contribute to reducing the adverse effects typically associated with their use.

**Fig. 3:**
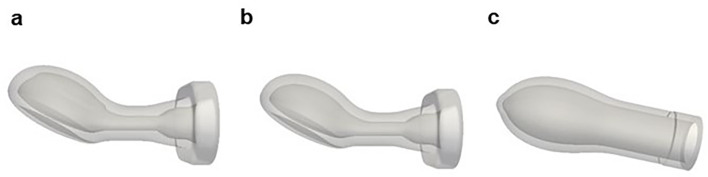
Nozzles of the different studied pacifiers employed for the computational models: **a**. Orthodontic Pacifier; **b**. Standard Pacifier; **c**. Conventional Pacifier

The parts of the pacifiers outside the oral cavity are not relevant for the computational model. Therefore, only the nozzle of each geometry was used for simulation purposes, as shown in Fig. 3.

Considering the methodology presented in the previous work [42], one computational model was created for each pacifier using the OpenFOAM^®^ open-source computational library [49], specifically the solids4Foam toolbox [50]. OpenFOAM^®^ is an open-source computational library developed by a large global community and has proven capable of handling complex multiphysical phenomena, thanks to its fast and versatile tools designed to solve a wide range of problems, including those addressed in this study.

### Pacifier effects comparison methods

As mentioned, malocclusion encompasses all types of misalignments of the teeth and maxillaries, including discrepancies in tooth dimensions and maxillary size, as well as poor relationships between dental arches in different anatomical planes and improper dental positioning [26]. To assess the likelihood of these issues, the maximum displacement of the six dental crowns considered was monitored across all computational models, and an OpenFOAM^®^ utility was developed to calculate the force on each dental crown.

Different pacifiers can also be compared based on the distribution of stresses on the palate and the force they exert on it.

### Tooth force algorithm

The utility developed to calculate the force exerted on dental crowns, *calculateForcesBetweenSets*, computes the force based on the cell zones defined in the computational mesh. These cell zones are established by a utility (*createCellSetsFromSTL*) that groups cells of the computational mesh using a closed 3D surface in STL format provided by the user. This information enables the identification of the computational mesh regions corresponding to the interface of different tissues, specifically the interface between two *cellZones*, one of which corresponds to the dental crown of interest.

The force exerted on each cell face at the cellZone interface near the given dental crown can then be calculated as follows:

1 $$\:{\varvec{F}}_{\varvec{i}}=\:{\varvec{\sigma\:}}_{\varvec{i}}\:\times\:{\varvec{A}}_{\varvec{i}},$$

where $\:{\varvec{\sigma\:}}_{\varvec{i}}$ is the stress vector calculated on the face, and $\:{A}_{i}\:$is the corresponding face area vector. The total force $\:{F}_{total}$ applied to each dental crown is the sum of the forces calculated on each face, computed across all faces at the interface, as follows:

2 $$\:{\varvec{F}}_{\varvec{t}\varvec{o}\varvec{t}\varvec{a}\varvec{l}}=\sum\:{\varvec{F}}_{\varvec{i}}$$

**Fig. 4:**
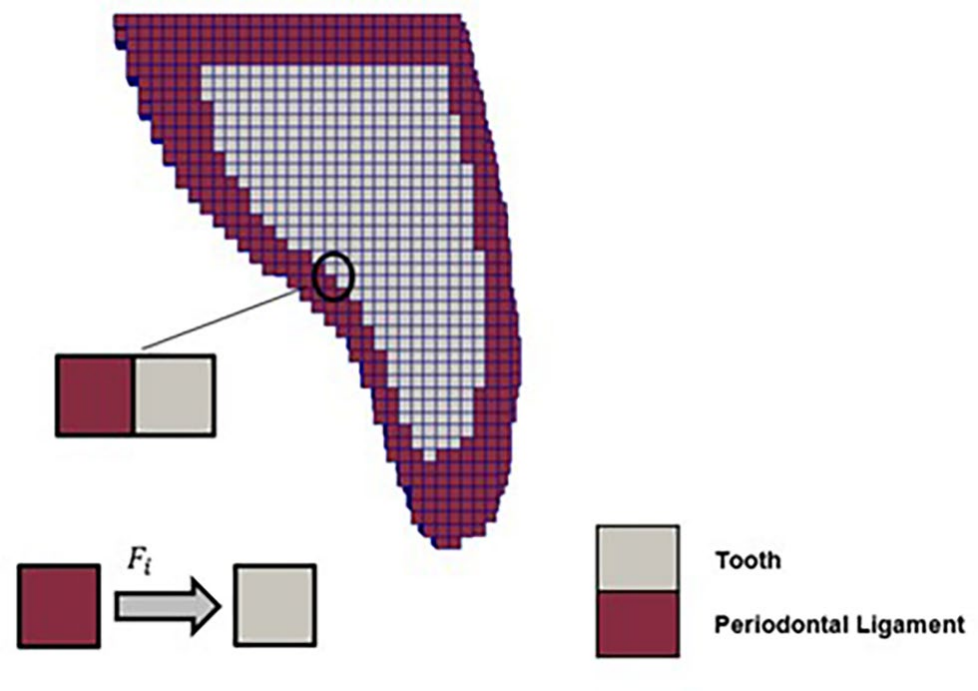
Section of the tooth mesh involved by the periodontal ligament and respective individual force calculation

Figure 4 shows the cut view of the computational mesh, where the computational cells for the dental tissue are surrounded by those of the periodontal ligament. Therefore, the force exerted on the dental tissue is calculated at the faces shared by the periodontal ligament and dental crown cells.

### Tooth displacement

The maximum displacement in each dental crown during two suction cycles also allows for comparing the effects of different pacifiers on the orofacial structures considered in the computational models prepared in this work.

The maximum displacement was calculated during the post-processing stage with the aid of ParaView software [51]. ParaView is a tool that allows visualizing and analyzing two-dimensional and three-dimensional datasets. It also includes features that allow calculating the maximum displacement of a set of computational cells over time. Therefore, for the different developed computational models, it was possible to obtain the evolution of the maximum displacement of the cells in a specific *cellZone*, which was used to calculate the maximum displacement evolution for each dental crown.

### Palate stress

In the developed computational models, the tongue moves, pushing the pacifier into contact with the palate. When the pacifier touches the palate, it exerts pressure that promotes deformation. Although pacifiers have the same mechanical properties, they differ in design, leading to varied geometric characteristics and shapes that are expected to induce different stress distributions on the palate’s tissues.

Contours of stress distributions on the palate were obtained during post-processing with ParaView, for each time step recorded during the calculation.

### Pacifier force

Since pressure on the palate is the force per unit area, the force exerted by the pacifier on the palate was calculated using the developed OpenFOAM^®^ utility - *calculateForcesOnPatches*. This utility calculates the force transmitted between different Cell Zones in contact, based on the numerical calculations performed by the *solids4Foam* utility.

## Results

In this work the model identified as the most realistic in [42] was used to obtain concrete results regarding the effects of different pacifiers. That model is part of a methodology that tests twelve different computational models to identify the most appropriate one for better understanding of the development of malocclusions. The analyses conducted in this work allow for comparing the effects of three different pacifier geometries: the orthodontic pacifier (OP), the standard pacifier (SP), and the conventional pacifier (CP).

The simulation studies comprise two suction cycles, during which different results were analyzed, namely: stress distribution on the palate surface, the evolution of the force exerted by the pacifier on the palate, the maximum displacement of the teeth, and the force exerted on the teeth.

### Palate stress

The analysis of stress distribution on the palate is clinically significant, as prolonged mechanical loading during suction may alter maxillary development, underscoring the implications of pacifier design on craniofacial growth.

**Fig. 5:**
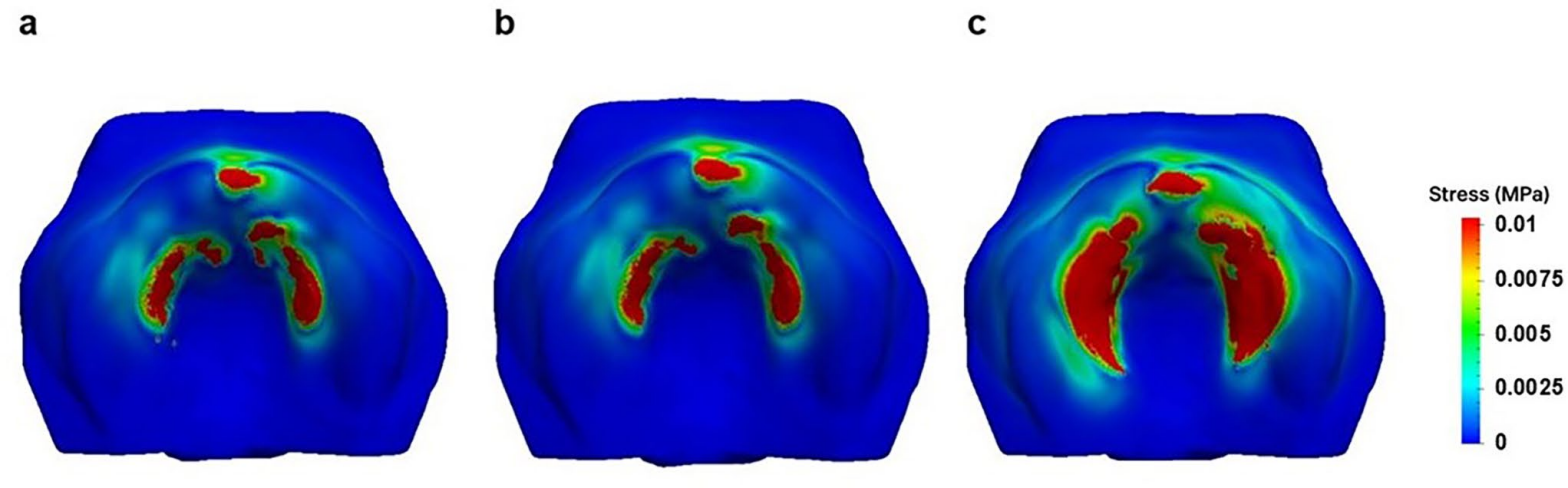
Stress distribution on the mucosa surface induced by the tested pacifiers: **a**. Orthodontic Pacifier; **b**. Standard Pacifier; **c**. Conventional Pacifier

**Fig. 6:**
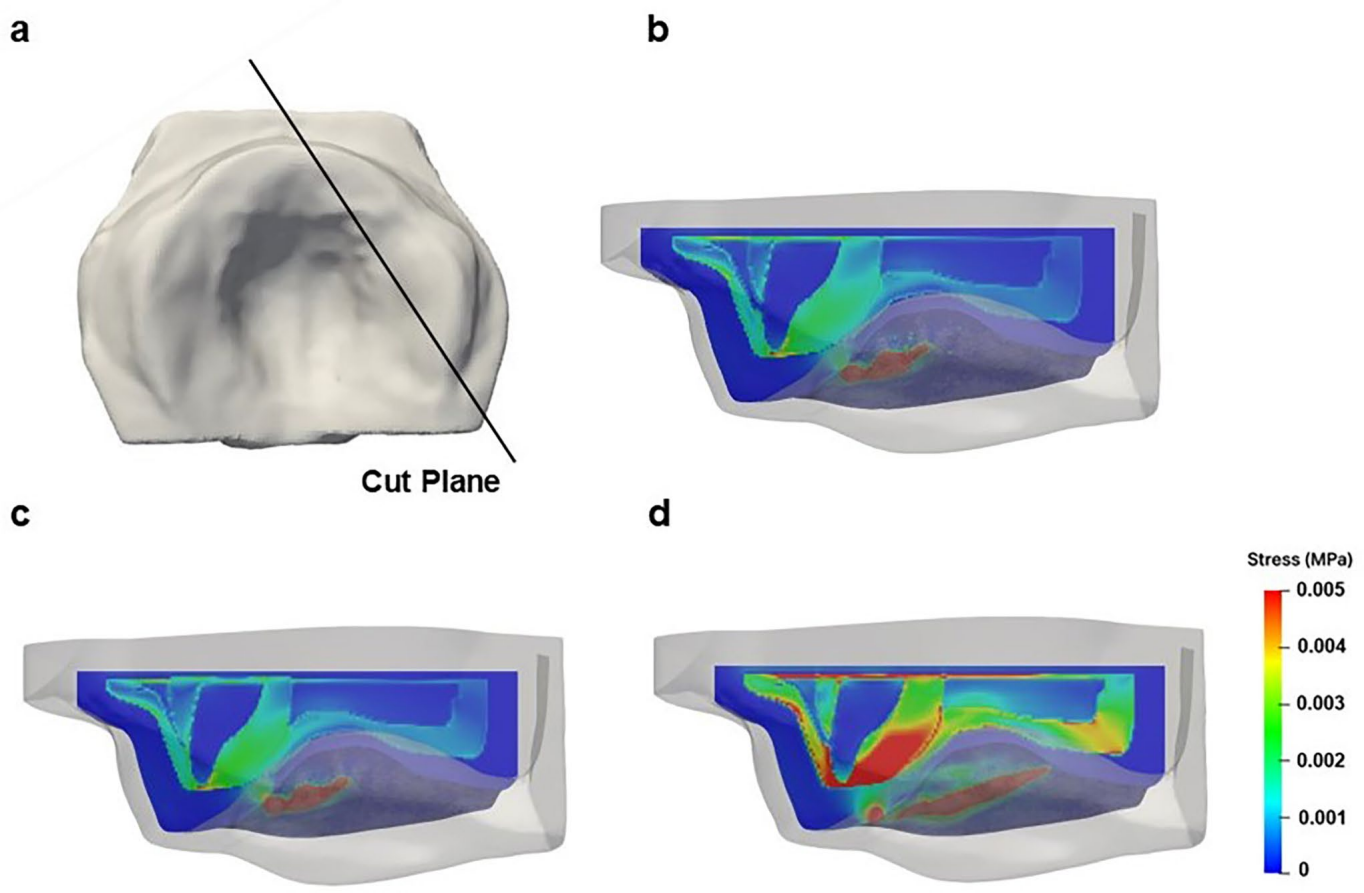
Cut view of the stress distribution in the internal tissues of the palate induced by the tested pacifiers: **a**. Cut plane; **b**. Orthodontic Pacifier; **c**. Standard Pacifier; **d**. Conventional Pacifier

The stress distribution on the palate for the three pacifier models is shown in Fig. 5, where the palate is presented in an occlusal projection, and the same scale was used for all distributions to facilitate comparison. Stress on the palate is mainly caused by tongue pressure pushing the pacifier upwards during suction. This simulation helps illustrate how different pacifier shapes distribute this pressure, which may contribute to malocclusion development. The results in Figs. 5 and 6 correspond to the moment when the tongue reaches its maximum displacement during the second peak, representing the peak of maximum Von Mises stress. Figure 5 displays a view of the palatal surface, where only the superficial distribution of maximum stress is visible. In contrast, Fig. 6 provides a cross-sectional view of the palate, allowing visualization of maximum stress distribution within the internal palatal tissues.

The results presented in Table 1 allow for a detailed comparison between the pacifier models, highlighting the biomechanical advantages of the ergonomic designs - Orthodontic Pacifier (OP) and Standard Pacifier (SP) - when compared to the Conventional Pacifier (CP).

Regarding stress distribution, OP and SP significantly reduced the volume of palatal mucosa exposed to von Mises Stress in the range of 0.01 to 0.05 MPa. Specifically, OP achieved a 95.70% reduction, while SP resulted in a 93.95% reduction, when compared to CP.

**Table 1:** Volume of mucosa (in mm³) exhibiting von mises stress values between 0.01 mpa and 0.05 mpa for each tested pacifier: orthodontic pacifier (OP), standard pacifier (SP), and conventional pacifier (CP); along with the percentage reduction in the volume of these maximum stress levels relative to the conventional pacifier

Threshold von Mises Stress (0.01 MPa to 0.05 MPa)				
Volume ($\:{\varvec{m}\varvec{m}}^{3})$			Decrease (%)	
OP	SP	CP	OP vs. CP	OP vs. SP
116,854	164,335	2717,4	95,70%	93,95%

### Pacifier force

The evaluation of forces exerted on the palate in crucial, as their magnitude and distribution during suction can influence maxillary development and lead to long-term craniofacial changes.

**Fig. 7:**
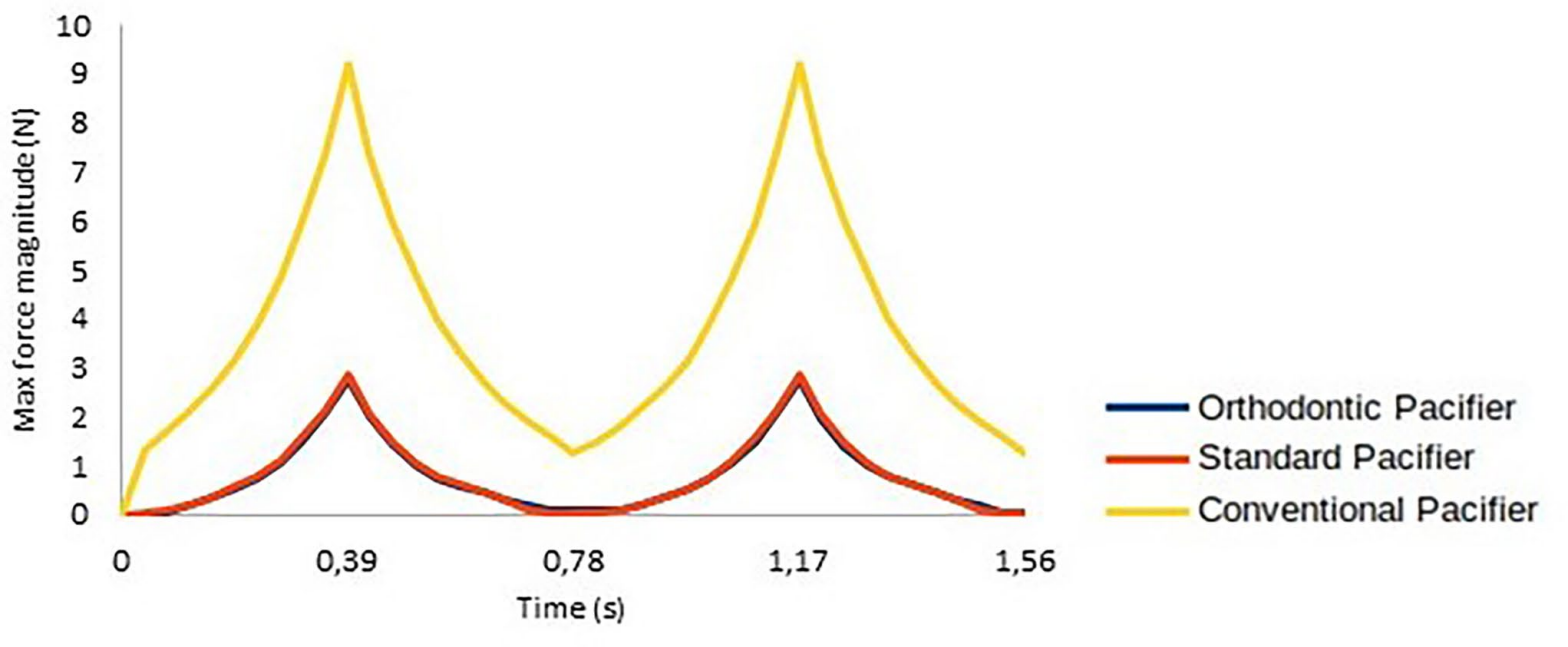
Evolution of the force magnitude exerted by the pacifiers on the palate over two suction cycles

The evolution of the force magnitude exerted by the pacifier on the palate during the two suction cycles was calculated for all tested computational models (OP, SP and CP models), with the results shown in Fig. 7.

### Tooth displacement

Tooth displacement during suction may indicate the force transferred to dental structures within the alveolar bone, a key factor in the misalignment of teeth during eruption.

**Fig. 8:**
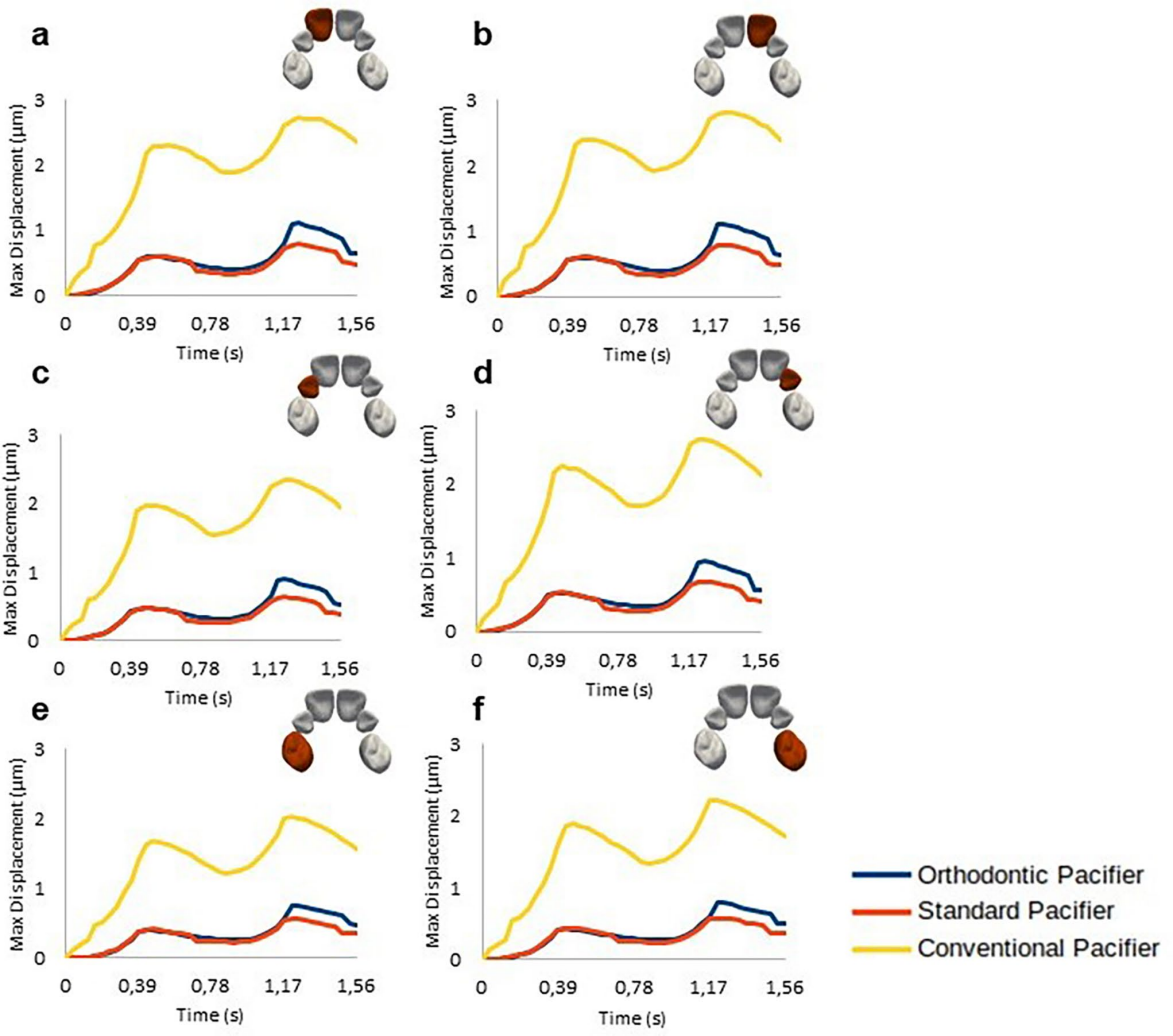
Evolution of the maximum displacement of dental crowns calculated over two suction cycles for **a**) Right Central Incisor; **b**) Left Central Incisor; **c**) Right Lateral Incisor; **d**) Left Lateral Incisor; **e**) Right First Molar; and **f**) Left First Molar

**Fig. 9:**
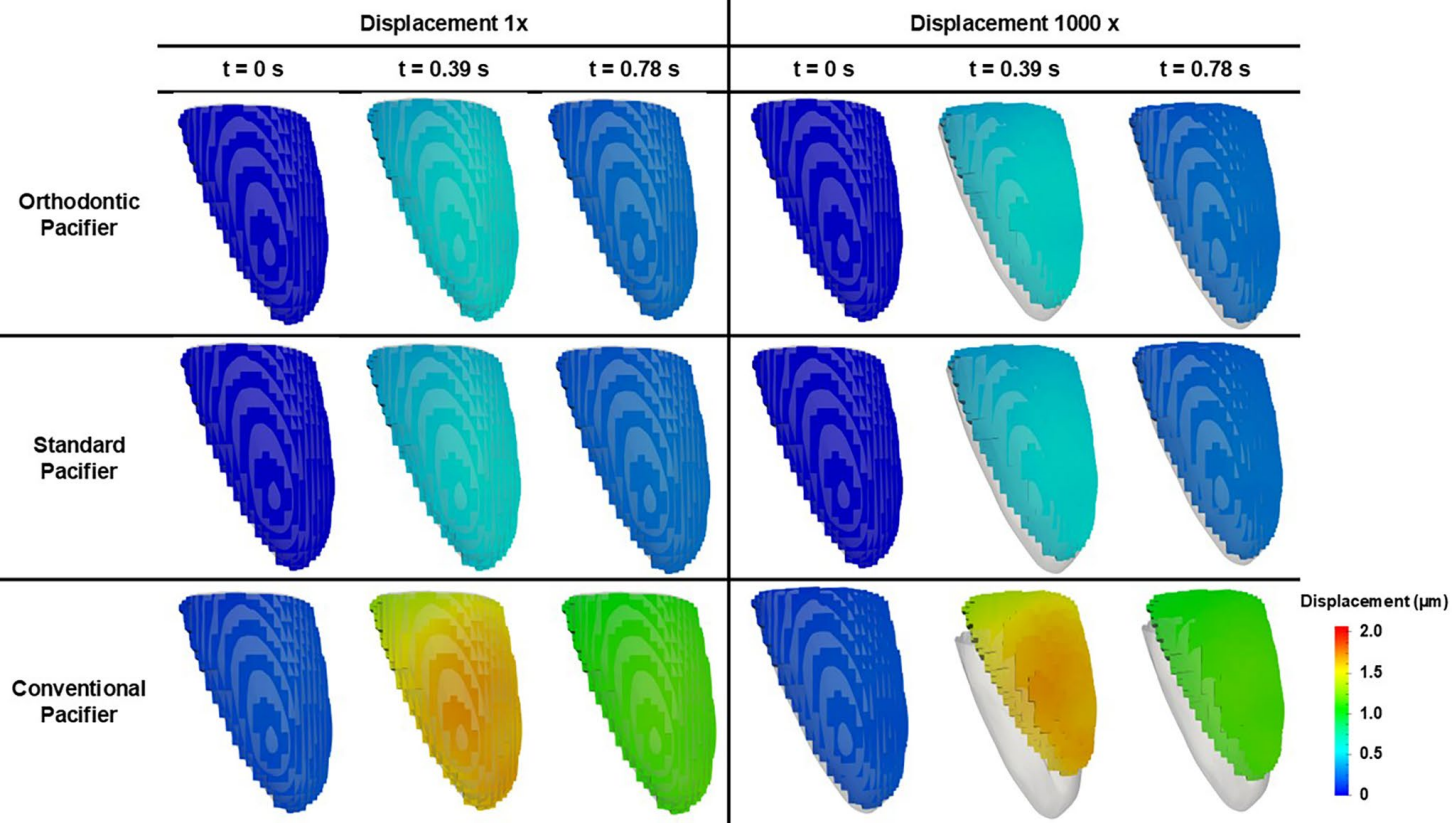
Maximum displacement of the crown of the central incisor in the three pacifiers models at 0, 0.39 and 0.78 s with scale factors of 1x and 1000x

The evolution of the maximum displacement of the six dental crowns during two suction cycles of the three different pacifiers is plotted in Fig. 8. Comparative results of the displacement distribution of the central incisors (teeth with a higher prevalence of malocclusions) for pacifier OP, SP, and CP models are illustrated in Fig. 9, which shows the states at three instants of time (0, 0.39 and 0.78 s) for the right central incisor crown across different pacifier models. Because real displacements are very small, results were also magnified 1000 times in the figure to clearly evidence the differences between pacifier models. These displacements may indicate tendencies for dental movement, which is relevant in early diagnosis of malocclusions.

Table 2 presents the displacement values (in micrometers) measured across all dental crowns for each pacifier model. The results indicate that both the OP and the SP significantly reduced tooth displacement compared to the CP. Specifically, the average percentage reduction in dental displacement for the SP ranged from 73 to 75%, while for the OP it ranged from 77 to 79%, relative to CP.

Furthermore, when comparing the two ergonomic models directly, the OP showed a greater reduction in displacement ranging from 15 to 17% compared to the SP.

**Table 2:**
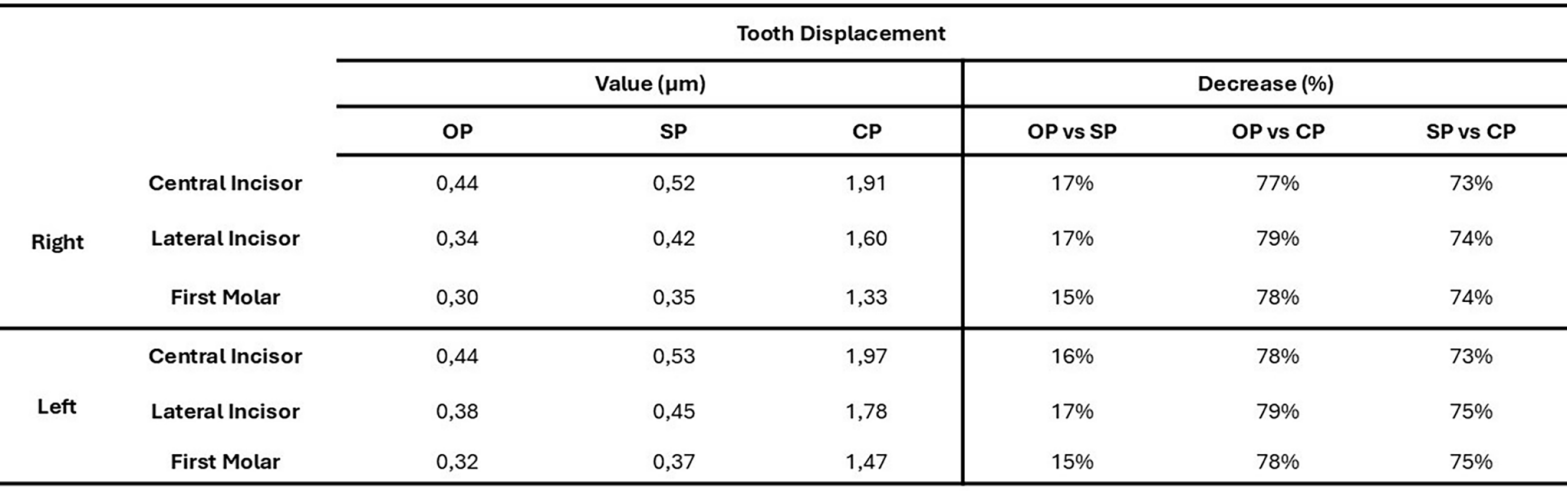
Displacement values (in µm) measured in each dental crown for the three pacifier models - Orthodontic pacifier (OP), standard pacifier (SP), and conventional pacifier (CP) - along with the percentage decrease in displacement for OP and SP models relative to the CP model

### Tooth force

The previously presented displacement of the dental crowns is caused by a force exerted on each dental crown, which in turn was generated by the pacifier’s pressure on the palate.

The forces transmitted to the teeth, when excessive or improperly distributed during suction, may contribute to malocclusions by affecting tooth positioning and the eruption process.

**Fig. 10:**
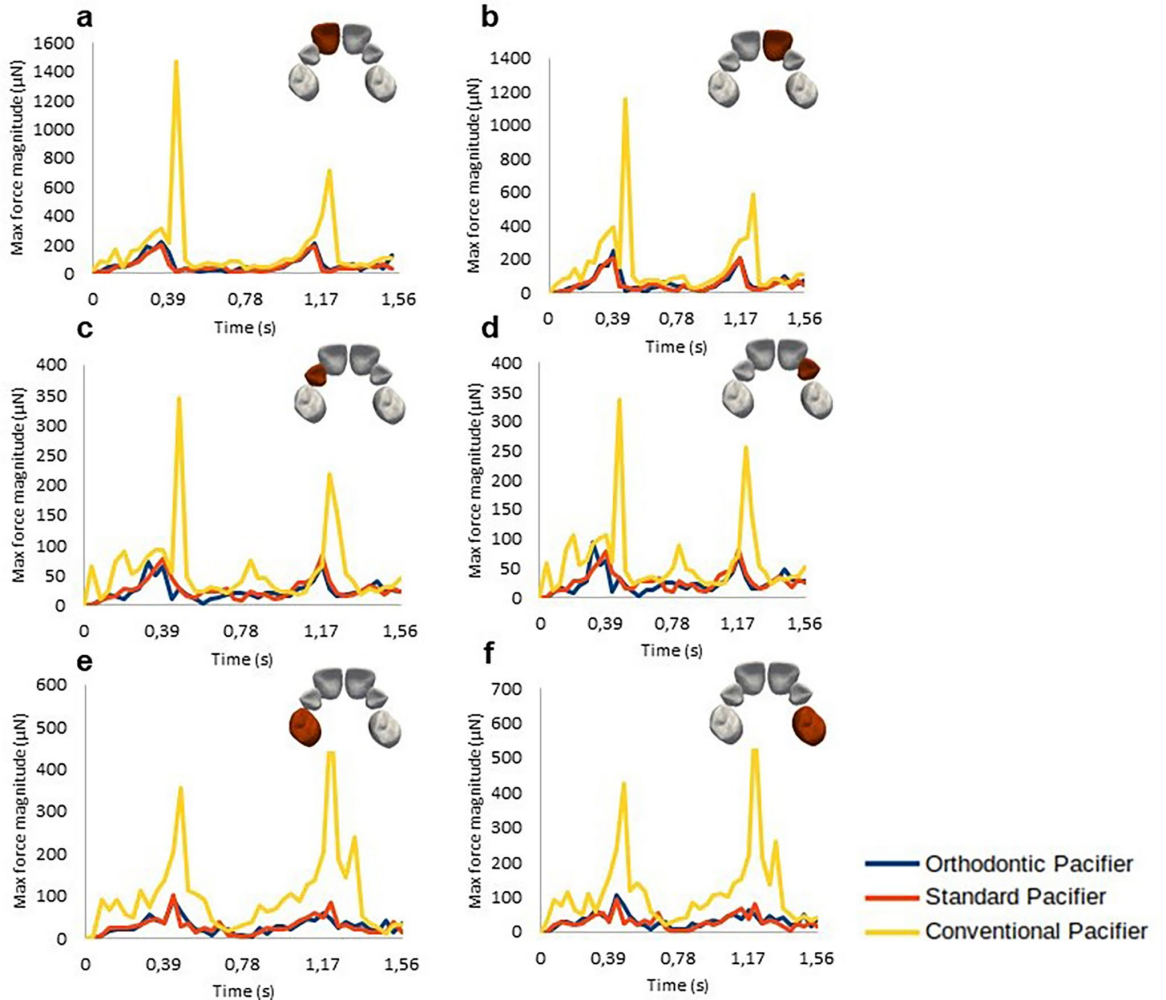
Evolution of the force magnitude exerted on all dental crowns over two suction cycles: **a**) Right Central Incisor; **b**) Left Central Incisor; **c**) Right Lateral Incisor; **d**) Left Lateral Incisor; **e**) Right First Molar; and **f**) Left First Molar

Fig. 10 presents the results of the evolution of the forces exerted on each dental crown for the OP, SP, and CP model

Table 3 presents the tooth force values (in µN) measured across all dental crowns for each pacifier model. The results indicate that both the OP and the SP significantly reduced the forces transmitted to the teeth compared to the CP. Specifically, the average percentage reduction in tooth force for the SP ranged from 59 to 72%, while for the OP it ranged from 54 to 75%, compared to the CP.

Furthermore, when directly comparing the two ergonomic models, the OP showed an additional reduction in tooth force of 3–11% compared to the SP.

**Table 3:**
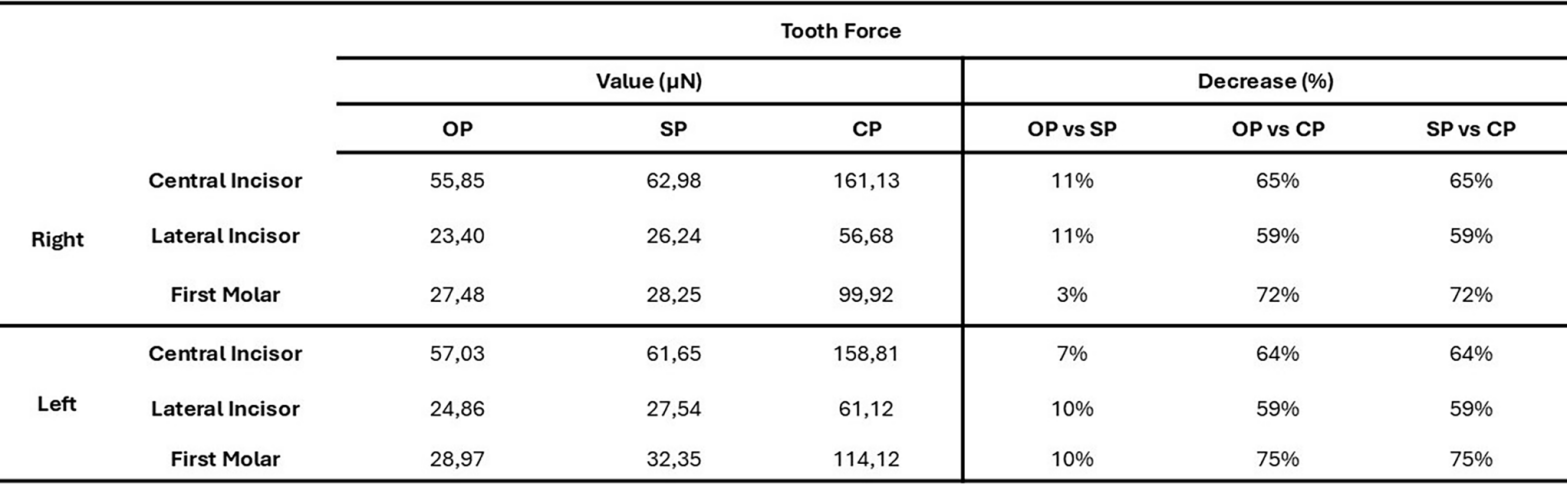
Force values (in µN) measured in each dental crown for the three pacifier models - Orthodontic pacifier (OP), standard pacifier (SP), and conventional pacifier (CP) - along with the percentage decrease in displacement for OP and SP models relative to the CP model

The results for the distribution of stress on the palate indicate that different pacifier geometries induce varying stress distributions and magnitudes. The CP shows significant differences compared to the OP and SP. This is because the CP deforms even at the lowest tongue position, exerting permanent stress on the palate, while the SP and OP adapt more naturally to the available space in the oral cavity.

Although the palate is nearly symmetrical with a U-shaped surface, it has some irregularities due to the gradual process of bone maturation in the orofacial structure. The round geometry of the CP leads to uneven peaks in the stress distribution. In contrast, the almost flat surfaces of the SP and OP conform more naturally to the palate, resulting in a smoother and more symmetrical stress distribution, with less pronounced peaks and a lower maximum value. Additionally, due to being forced at the lowest tongue position, the stress magnitude near the central incisors for the CP is higher than that exerted by the OP and SP (See Fig. 10).

## Discussion

The conclusions about the stress distribution on the palatal surface are supported by the evolution of force magnitude illustrated in Fig. 10, which shows significant differences among the various pacifiers. These results demonstrate that, for all pacifier models, the CP exerts a much greater force over two suction cycles compared to the SP and OP, while the effects of SP and OP are similar. This can be attributed to the geometric similarity between OP and SP, with the main difference being the reduced thickness of the OP in the area in contact with the palate. The results indicate that this difference in thickness is not sufficient to impact stress/force distribution on the palate (Figs. 5 and 6). However, as observed in Fig. 6, the CP model exhibits significantly higher stress concentration around the central incisor, indicating greater tissue deformation adjacent to the tooth compared to the OP and SP models. This suggests that the continuous palatal coverage configuration may induce higher stress levels in the peri-dental tissues, possibly due to the way force is distributed in that region.

Stress distribution analysis revealed that both the OP and SP substantially reduced the volume of palatal mucosa exposed to von Mises stress in the range of 0.05 to 0.01 MPa. The OP model demonstrated a 95.70% reduction, while the SP model showed a 93.95% reduction compared to the CP (Table 1). These results indicate that ergonomic features, such as anatomical conformity and improved stress distribution across the palate, significantly reduce the mechanical load on palatal tissues.

In clinical practice, the higher stress concentration observed with the CP suggests a greater risk of interference in the transverse growth of the palate and in tooth positioning (Fig. 6). This may lead to adverse morphological changes in orofacial development (posterior cross bite), especially if use is prolonged during critical growth periods.

Further information on the effects of different pacifier geometries on orofacial structures can be obtained by analyzing the von Mises stress peak results and the maximum forces exerted on the dental crowns (Fig. 10), which consequently lead to their displacement (Figs. 8 and 9). These results indicate that pacifiers have varying effects on dental tissues. Specifically, the CP causes greater maximum displacement than the OP and SP, while the maximum displacement in dental crowns caused by the latter two pacifiers is similar.

According to Table 3, both ergonomic models significantly reduced the forces transmitted to the teeth, with reductions ranging from 59 to 72% for the SP and from 54 to 75% for the OP compared to the CP. In a direct comparison between the two ergonomic models, the OP demonstrated superior performance, with an additional reduction in dental forces ranging from 3 to 11%. Likewise, Table 2 shows that dental displacement was considerably lower in the OP and SP models compared to the CP, with reductions of 77–79% for the OP and 73–75% for the SP. Notably, the OP outperformed the SP, presenting an additional reduction in displacement of 15–17%, further reinforcing its potential to minimize undesired tooth movement during pacifier use.

This displacement pattern correlates strongly with clinical observations in children with a history of prolonged use of conventional pacifiers, where there is an increased prevalence of anterior open bite (AOB) and anterior teeth protrusion (PCB), indicating a direct impact on orofacial development.

Analysis of the results for each dental crown reveals that the central incisors exhibit higher displacement and force magnitude compared to other crowns (Fig. 10). This may be attributed to CP’s lack of conformity to the natural shape of the palate, concentrating its effects in the central region where the central incisors erupt.

Previous clinical studies [11, 14, 43, 52, 53] have reported an association between prolonged use of conventional pacifiers and changes in the inclination of the central incisors, as well as alterations in palatal morphology. The stress and deformation patterns observed in this study (Figs. 5 and 6) support these findings, particularly regarding the increased force applied to the central incisors by the conventional pacifier model (CP). Additionally, recent clinical data from a randomized controlled trial [52] further emphasizes the role of pacifier design in the development of malocclusions. Although that study did not find statistically significant differences in overjet or anterior open bite between the evaluated groups, it referenced other investigations [43, 53] that reported a higher prevalence of these occlusal alterations among users of conventional pacifiers. These findings highlight the importance of anatomical pacifier design as a preventive strategy to minimize adverse effects on anterior dentition.

The results indicate that the CP promotes higher stress distribution compared to the other analyzed pacifiers (OP and SP). Consequently, the CP exerts greater force on the palate than the OP and SP, making it more likely to cause misalignment of the dental arches and an increase in palatal depth.

Although the simulation results indicate a higher risk of AOB and PCB, it is important to clarify that malocclusions are a multifactorial condition, and several individual parameters should be considered, including breathing and sucking patterns regarding duration, frequency and intensity of use.

The maximum force and displacement calculated for each dental crown suggest that the CP significantly increases the likelihood of malocclusions compared to the OP and SP. Specifically, the CP resulted in greater displacement of the central incisor crowns (Figs. 8 and 9), indicating a higher risk of anterior open bite (AOB), Class I malocclusions with protrusion of the central incisors, and Class II malocclusions.

Previous studies employing finite element analysis and computational simulations [32–36] have consistently shown that pacifier geometry significantly influences the distribution of mechanical stress on the palate. These studies concluded that anatomically designed or orthodontic pacifiers tend to reduce peak stress concentrations and better mimic natural oral structures, promoting healthier oral development [32, 33, 35, 36].

The present study corroborates these findings, as the observed force distribution patterns align with those previously reported. Notably, our model advances prior research by incorporating two full suction cycles (Fig. 7) and a more anatomically detailed representation of the infant palate, enhancing the physiological accuracy and clinical relevance of the simulations.

Nonetheless, it is necessary to acknowledge that this methodology has limitations, such as the absence of the complexity of tongue muscle movement during suction and other anatomical simplifications based on literature rather than clinical data. Future studies should aim to validate these results through comparisons with clinical images or physical models from patients.

Finally, the results demonstrate that the developed computational methodology is effective for comparing the effects of different pacifier geometries and for providing concrete data that quantify the influence of non-nutritive sucking habits on the development of dental arch misalignment and, consequently, the formation of malocclusions.

It is important to emphasize that, although computational models allow controlled analysis of forces and stresses, they do not fully replicate the variables of a longitudinal clinical study. However, the data presented here provides an objective basis that can contribute to the development of pacifiers with more ergonomic designs and suitable materials, reducing the risks of dentofacial changes and promoting the use of safer and healthier products.

## Conclusions

This study highlights the significant influence of pacifier geometry on the development of orofacial structures, demonstrating that different designs lead to distinct patterns of stress distribution on the palate and tooth displacement. The developed computational methodology proved to be a robust tool, capable of accurately quantifying the effects of pacifiers and providing valuable insights into the impact of non-nutritive sucking habits on malocclusions formation.

The findings emphasize the importance of carefully considering pacifier design, prioritizing shapes that minimize excessive pressure on developing oral structures, while also preserving potential functional benefits such as stimulating orofacial muscles. Moreover, the research underscores the need to limit the duration and intensity of pacifier use to mitigate potential adverse effects.

In the future, these results can support the improvement of pacifier design, contributing to the development of more ergonomic and safer models that combine comfort for children and aid healthy oral development when their use is indicated.

## Electronic supplementary material

Below is the link to the electronic supplementary material.

Supplementary Material 1

## Data Availability

No datasets were generated or analysed during the current study.

## Abbreviations

3D: Three-Dimensional
AOB: Anterior Open Bite
CP: Conventional Pacifier
OpenFOAM: Open Field Operation and Manipulation
OP: Orthodontic Pacifier
PCB: Posterior Cross Bite
SP: Standard Pacifier
STL: Stereolithography
