# Supplementary material for: Effect of different pacifier designs on orofacial tissues: a computational simulation comparative study
Source: Clin Oral Investig. 2025 Jun 25;29(7):356. doi: 10.1007/s00784-025-06428-9 (PMC12198337; doi:10.1007/s00784-025-06428-9)
Supplement: Supplementary file 1 — Supplementary Material 1 [file 784_2025_6428_MOESM1_ESM.docx]

**Supplementary Information**

**Detailed Methodology for the Development of the Computational Model to Assess Pacifier Design**

*Detailed Methodology published in Pereira et al. 2024*

**Simulation Framework Summary**

Numerical simulations were performed using OpenFOAM® [50], an open-source finite volume platform, enhanced with the solids4Foam toolbox [51] for solid mechanics analysis. This setup supports simulations involving small and large deformations, elastic-plastic behavior, various constitutive models, and complex boundary conditions. The finite volume discretization ensures accurate results on unstructured meshes. The framework was chosen for its flexibility, customizability, and strong community support.

**1. Pre-processing Tools**

- **Blender™ [49] –** Used for detailed 3D modeling of anatomical structures, including the palate (mucosa, cortical bone, cancellous bone, alveolar bone, periodontal ligament, teeth), pacifier, and tongue.
- **cfMesh [55] –** Automated mesh generation using Cartesian and STL-based approaches, suitable for complex geometries.
- **mergeMeshes (OpenFOAM) –** Combines individual component meshes (palate, pacifier and tongue) into a unified computational domain.
- **setMatFromCellZones (solids4Foam) –** Assigns material properties to predefined cell zones for a heterogeneous computational model.

**2. Post-processing Tool**

- **ParaView [52] –** Used for visualizing and analyzing stress distributions, forces exerted on orofacial structures, and displacement fields.

**3. Custom Utilities Developed**

- **createCellSetsFromSTL –** Selects specific regions within the mesh based on STL geometry inputs the mechanical properties of different tissues and structures.
- **calculateForcesBetweenSets** – Computes interface forces between specified cell zones, aiding the analysis of contact forces, such as those exerted on dental crowns.

**2. Geometrical Model Construction**

**2.1 Palate**

**Source:** The palate geometry was obtained from a high-resolution 3D scan of a plaster model (Fig. 1) of a 6-month-old infant’s maxillary arch, using the **NobelProcera® 2G** optical scanner. The scan, exported in **STL format**, preserved anatomical detail and was post-processed for watertightness and mesh compatibility.


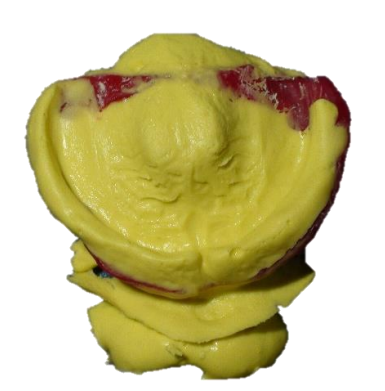


**Fig. 1.** Plaster Model

**Tissues represented:** Mucosa**,** Cortical bone**,** Cancellous bone**,** Alveolar bone**,** Periodontal ligament and Six primary teeth.

**Periodontal ligament and Tooth Specifications (Fig. 2):**

- 1. Six primary teeth (central and lateral incisors, first molars)
  2. Surrounding periodontal ligament (1 mm thickness)
  3. Embedded in alveolar bone


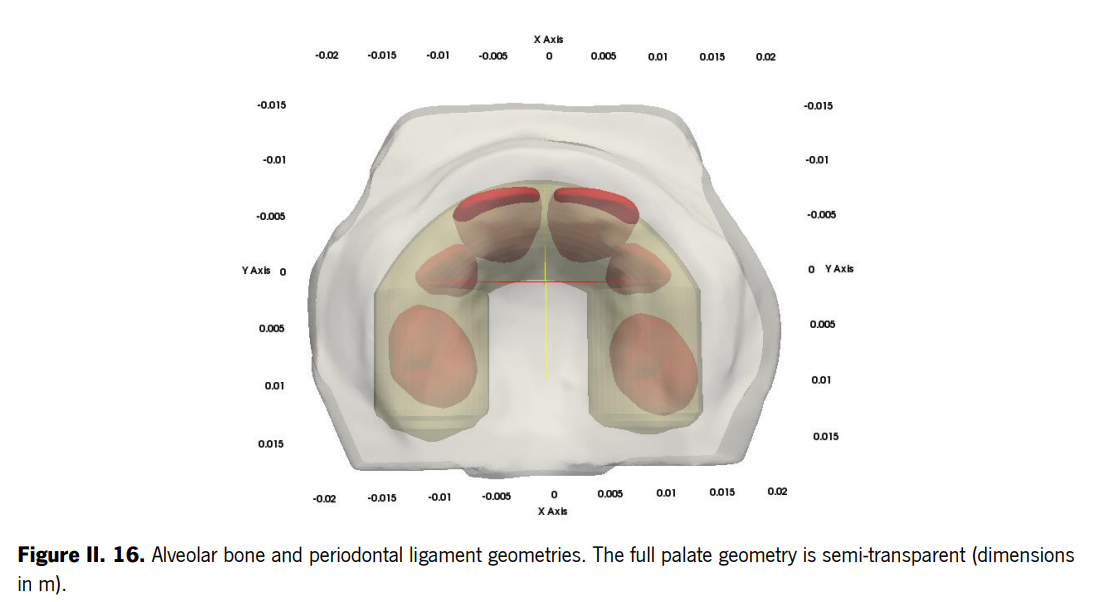


**Fig. 2** Alveolar bone and periodontal ligament geometries. The full palate geometry is semi-transparent (dimensions in m).

**2.2 Pacifier**

**Previous work [42]**

- **Model:** NUK Genius Orthodontic, Size 1 (for 6-month-old infants)
- **Source:** CAD file provided by manufacturer (Mapa GmbH)

**Current work**

- **Orthodontic Model:** NUK Genius Orthodontic, Size 1 (for 6-month-old infants) – STL file provided by manufacturer (Mapa GmbH) (Fig. 3).
- **Standard Model:** NUK Standard, Size 1 (for 6-month-old infants) – STL file provided by manufacturer (Mapa GmbH) (Fig. 4).
- **Conventional Model:** Based on the NUK Standard, Size 1 pacifier (for 6-month-old infants). Dure to the unavailability of and STL file from the manufacturer, the geometry was manually reconstructed in Blender™ using reference images and dimensions obtained from the physical product. The modeling process was conducted with a focus on preserving the overall shape, proportions, and design integrity of the original pacifier to ensure a realistic representation suitable for simulation purposes (Fig. 4 and Fig. 5).


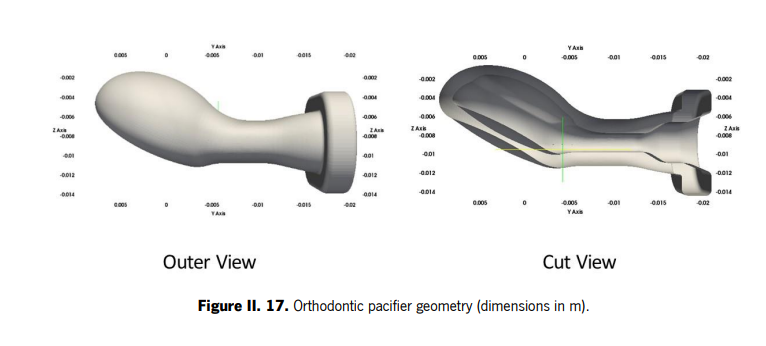


**Fig. 3** Orthodontic pacifier geometric (dimensions in mm)


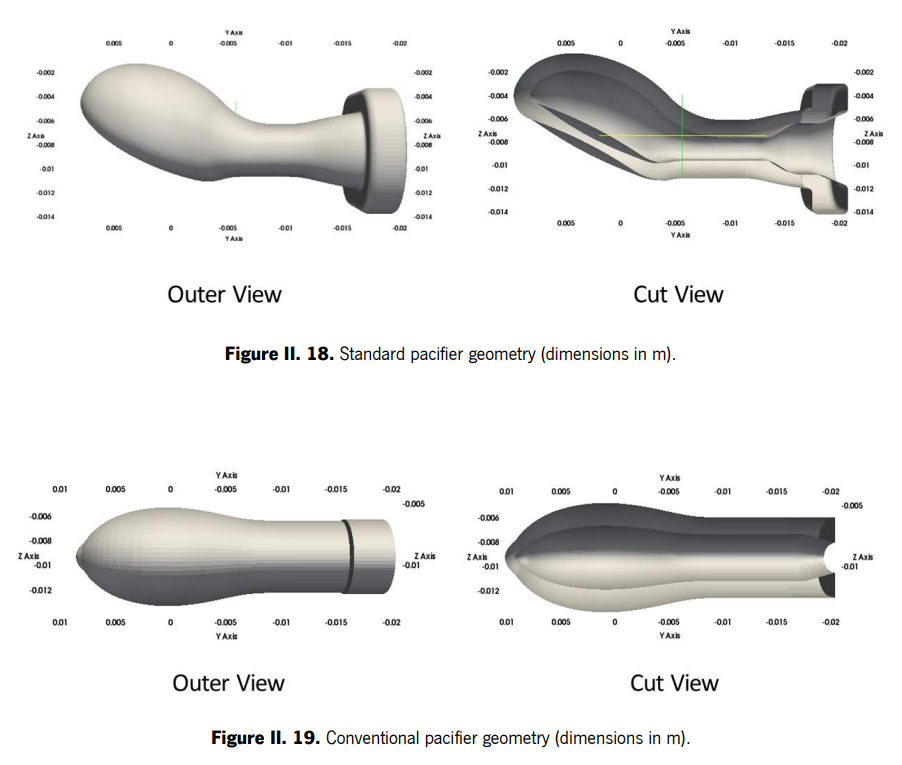


**Fig. 3** Standard pacifier (SP) geometric (dimensions in mm)


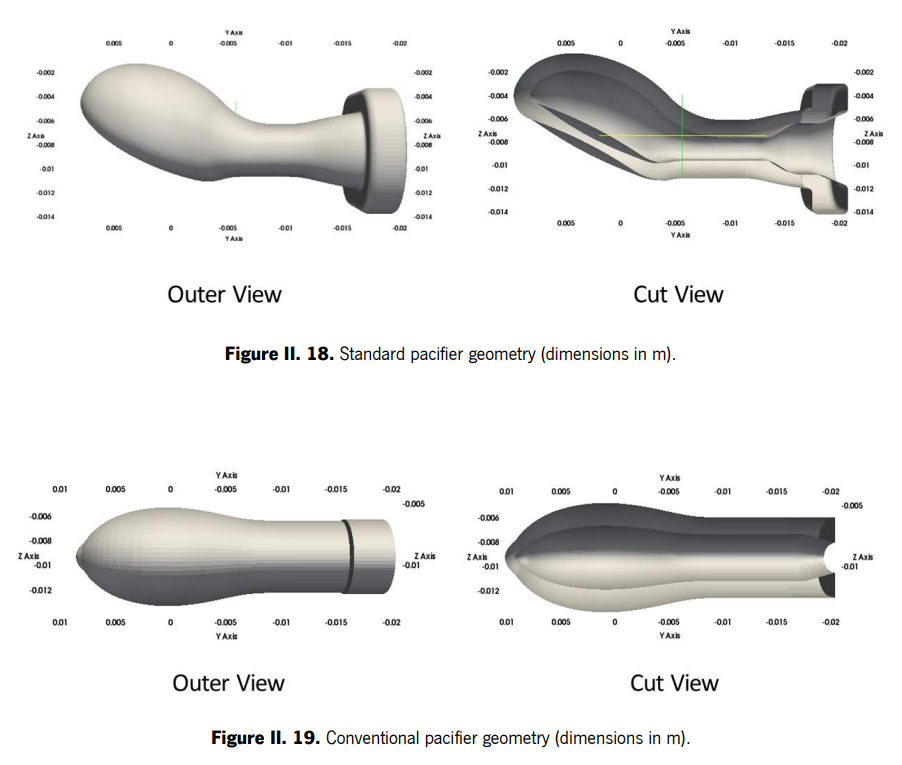


**Fig. 4** Conventional pacifier (CP) geometric (dimensions in mm)

**2.3 Tongue**

**Dimensions:** 3.74 cm (length) × 1.69 cm (width) (Fig. 5)

**Muscle Zones Included:** *Genioglossus*, *Geniohyoid*, *Hyoglossus*, and *Styloglossus* (approximated based on anatomical positioning) (Fig. 5).

**Motion:** The tongue was subjected to cyclic vertical displacement throughout the simulation, replicating the cyclic motion associated with infant suction behavior.


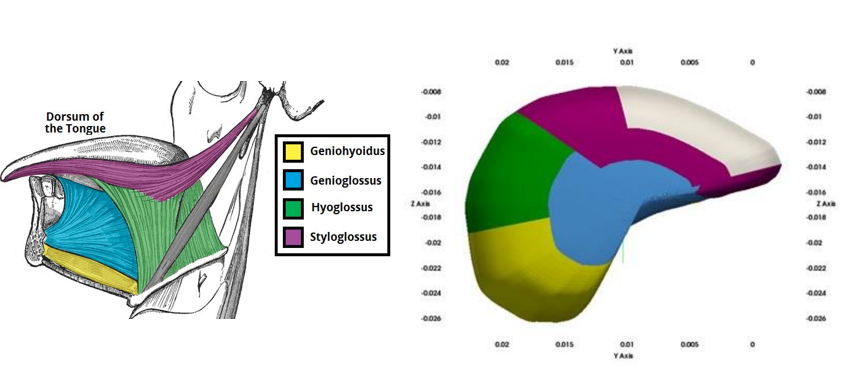


**Fig. 5** Tongue with anatomical representation of the muscles (right) and respective 3D model with dimensions in m (left)

**3. Mesh Details**

**Tool:** cfMesh [55] (CartesianMesh workflow)

**Mesh Size:** Approximately 2.2 million cells (merged for palate, tongue, and pacifier)

The mesh utilized in the simulations incorporates detailed segmentation of individual anatomical tissues within the combined computational domain.

**4. Tissue Segmentation and Material Properties**

Each anatomical region was assigned specific mechanical properties using the custom utility *setMatFromCellZones*. All tissues were modeled as linearly elastic. The mechanical parameters for each tissue type were derived from values reported in the literature and are detailed in **Supplementary Material 3** of Pereira et al. [42].

**5. Boundary Conditions**

The model is divided into distinct **boundary surface zones**, each corresponding to anatomical or functional regions relevant to the simulation. These zones are used to apply specific **mechanical boundary conditions**, including fixed constraints and prescribed displacements. A vertical cyclic displacement (maximum of 4 mm) is applied to the Genioglossus Tongue Muscle to simulate suction motion.

Each surface was defined and labeled based on anatomical landmarks and simulation requirements, allowing for targeted application of loads and constraints for realistic mechanical behavior. These assignments are illustrated in the schematic Fig.6.


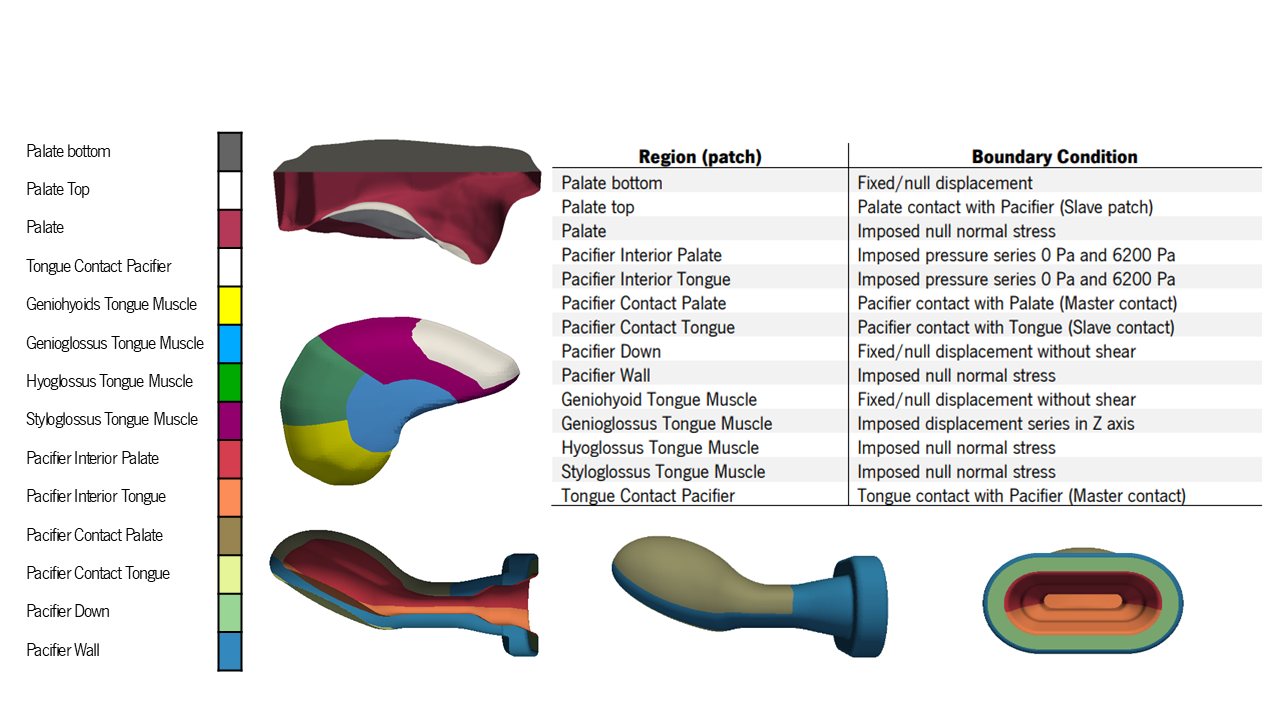


**Fig. 6** Representation of defined patches that have been assigned certain boundary conditions and boundary conditions description of the entire computational model developed.

**6. Model Variants**

In Pereira et al. [42], the models were categorized by the number and type of tissues included: the **1T model** consists of a single homogeneous tissue and serves as a baseline reference from the literature; the **3T model** includes mucosa, cortical bone, and cancellous bone; the **4T model** adds alveolar bone; and the **5T model**, the most detailed and realistic, incorporates all previous tissues along with the periodontal ligament (See Fig. 7)


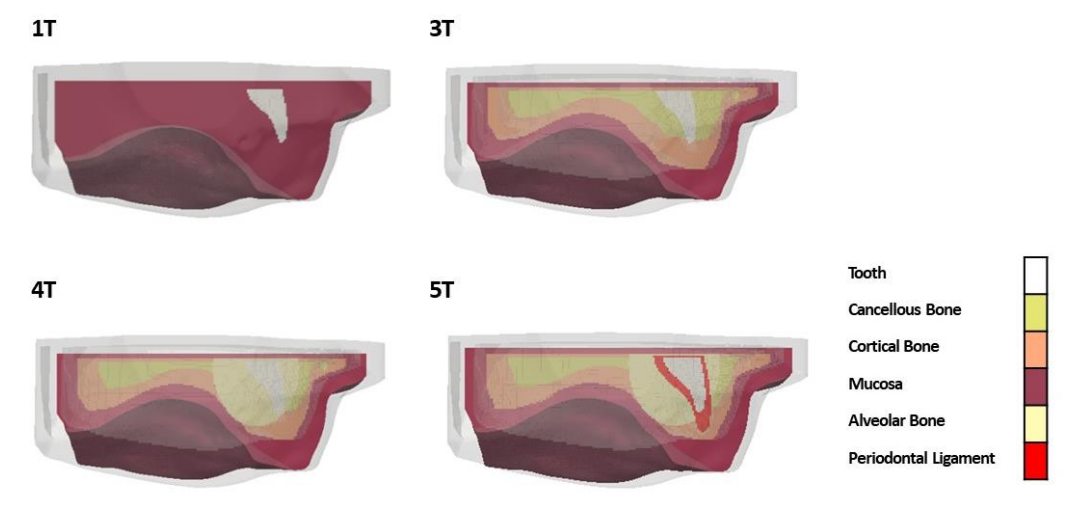


**Fig 7.** Cross-sectional images of Models 1T to 5T

**7. Model Selection to Pacifier Comparison Method**

In previous work [42], the selection of the most suitable model for analyzing the interaction between pacifiers and oral structures relied on a comparative approach with varying levels of tissue complexity, represented by models 1T to 5T (Fig. 7). Each model progressively incorporated more anatomically relevant tissues, starting with a homogeneous single-tissue representation (1T) and advancing to include mucosa, cortical bone, cancellous bone, alveolar bone, and finally the periodontal ligament in model 5T.

The analysis showed that the more detailed models, particularly model 5T, accurately reproduced the distributions of stress, displacement, and reaction forces at the interface between the pacifiers and oral tissues. Model 5T stood out for including the periodontal ligament, a key structure in dental load distribution, which significantly altered the mechanical response patterns compared to simplified models. This realistic response to simulated loading was essential for accurately assessing the impact of different pacifier designs. Therefore, model 5T was deemed the most appropriate for representing real clinical conditions and for providing reliable data to support comparisons between different pacifier designs in the present study.

**For more details on the computational methodology, please refer to manuscript reference [42]:**

*Pereira R, Romero J, Norton A, et al (2024) Advancing the assessment of pacifier effects with a novel computational method. BMC Oral Health 24:87.* [*https://doi.org/10.1186/s12903-023-03848-5*](https://doi.org/10.1186/s12903-023-03848-5)*.*
